# Low Sense of Coherence (SOC) is a mirror of general anxiety and persistent depressive symptoms in adolescent girls - a cross-sectional study of a clinical and a non-clinical cohort

**DOI:** 10.1186/1477-7525-8-58

**Published:** 2010-06-10

**Authors:** Eva C Henje Blom, Eva Serlachius, Jan-Olov Larsson, Töres Theorell, Martin Ingvar

**Affiliations:** 1 Department of Clinical Neuroscience, Karolinska Institutet, Sweden; 2 Department of Woman and Child Health, Karolinska Institutet, Sweden; 3 The Stress Research Institute, Stockholm University, Sweden

## Abstract

**Background:**

The Sense of Coherence (SOC) scale is assumed to measure a distinct salutogenic construct separated from measures of anxiety and depression. Our aim was to challenge this concept.

**Methods:**

The SOC-scale, Beck's Depression Inventory (BDI), Beck's Anxiety Inventory (BAI) , the emotional subscale of the Strengths and Difficulties Questionnaire (SDQ-em) and self-assessed health-related and physiological parameters were collected from a sample of non-clinical adolescent females (n = 66, mean age 16.5 years with a range of 15.9-17.7 years) and from female psychiatric patients (n = 73), mean age 16.8 years with a range of 14.5-18.4 years), with diagnoses of major depressive disorders (MDD) and anxiety disorders.

**Results:**

The SOC scores showed high inverse correlations to BDI, BAI and SDQ-em. In the non-clinical sample the correlation coefficient was -0.86 to -0.73 and in the clinical samples -0.74 to -0.53 (p < 0.001). Multiple regression models showed that BDI was the strongest predictor of SOC in the non-clinical (beta coefficient -0.47) and clinical sample (beta coefficient -0.52). The total degree of explanation of self assessed anxiety and depression on the SOC variance estimated by multiple R^2 ^= 0.74, adjusted R^2 ^= 0.73 in the non-clinical sample and multiple R^2 ^= 0.66, adjusted R^2 ^= 0.65 in the clinical sample.

Multivariate analyses failed to isolate SOC as a separate construct and the SOC-scale, BDI, BAI and SDQ-em showed similar patterns of correlations to self-reported and physiological health parameters in both samples. The SOC-scale was the most stable measure over six months.

**Conclusions:**

The SOC-scale did not appear to be a measure of a distinct salutogenic construct, but an inverse measure of persistent depressive symptoms and generalized social anxiety similar to the diagnostic criteria for major depressive disorder (MDD), dysthymic disorder, generalized anxiety disorder (GAD) or generalized social anxiety disorder (SAD) according to DSM-IV. These symptoms were better captured with SOC than by the specialized scales for anxiety and depression. Self-assessment scales that adequately identify MDD, dysthymic disorder, GAD and SAD need to be implemented. Comorbidity of these disorders is common in adolescent females and corresponds to a more severe symptomatology and impaired global function.

## Introduction

The Sense of Coherence (SOC) construct is based on Antonovsky's salutogenic theory in which protective and risk factors were considered to be qualitatively and dimensionally different [1]. Antonovsky designed a Sense of Coherence scale with 29 items and hypothesized that the SOC scale specifically measured three protective factors together constituting a global salutogenic factor: 1. the extent to which individuals are likely to perceive stressors as predictable and explicable (comprehensibility), 2. the extent to which they have confidence in their capacity to overcome the stressors (manageability) and 3. the extent to which they judge it worthwhile to take on the challenge (meaningfulness) [1]. High SOC was suggested to mirror a successful coping with stressors and thereby increase resilience. Later studies by Antonovsky himself and others have concluded that the SOC scale seemed to be a reliable, valid, and cross culturally applicable measure of how people cope with stressful situations and stay well [2,3].While the predictive power of SOC in relation to psychological health has been confirmed [4], the predictive power on physical health is still a matter of debate [5,6].

The discriminative validity of the SOC scale in relation to measures of depression and anxiety has been questioned [7]. Strong negative correlations have been found between SOC scores and measures of depression and anxiety in adults [8]. Physiological health parameters such as body mass index, blood pressure and saliva cortisol correlate in a similar way to SOC and measures of anxiety and depression [9,10].

The association between SOC and symptoms of anxiety and depression may apply to teenagers as well [11]. A high SOC score has been suggested to buffer the negative impact of emotion-oriented coping on suicidal manifestation in adolescent girls [12]. It is clinically important to establish the independence of SOC in relation to measures of anxiety and depression especially in the young. It is well known that symptoms of anxiety and depression early in life are risk factors for future psychiatric problems and the absence of these symptoms may be important salutogenic factors expressed by a high sense of coherence. If a low sense of coherence simply mirrors anxious and depressive problems, evidence-based methods for treatment may prevent chronic development and enhance the individual's general resistance resources.

Antonovsky mentions that the social environment is an important factor in forming the SOC [1]. Psychological symptoms and abuse in childhood also seem to influence the individual SOC [13,14]. From young adulthood SOC was assumed to have stabilized and show fluctuations of only about ten percent, except when faced with major life changes. According to the suggested model individuals with a strong SOC would show less variability of SOC over time [3]. The literature is inconclusive regarding temporal stability of SOC and whether SOC really is a trait measure as suggested by Antonovsky. Recent data imply a stabilization of SOC already at age 15 [15], but contradictory to Antonovsky's statement it has also been reported that SOC increases with age [10,16,17]. Furthermore, epidemiological data show that changes of SOC are related to societal changes and psychiatric complaints in the population [18]. and interventions with mindfulness based stress reduction lead to an increase of SOC scores [19], which implies that SOC is rather a state measure.

Teenage girls show increased vulnerability to anxiety disorders and depression compared to boys [20,21]. The gender differences apply also for SOC. Both teenage and adult females have weaker SOC than men [10,13,22]. The SOC scale, like psychiatric self-assessment scales, is used in the same versions for boys and girls without adaption to gender. In order to limit the variability of the sample we chose to focus solely on adolescent girls.

The factors suggested in forming SOC may also be applicable as risk factors for future development of anxiety and depression. It is well known that symptoms of depression and anxiety in childhood and adolescence have a negative impact on future health [23-25]. It is important to elucidate whether the SOC scale measures specific protective abilities that can be identified and targeted for training - or if the focus should be identification and treatment of depression and anxiety in this age group.

The aim of the present study was to challenge the concept of SOC as a distinct salutogenic construct separated from measures of anxiety and depression. In this paper we explore in depth the SOC construct based on data from a cohort study, in which we noted that the ability of SOC to discriminate caseness of anxiety disorders (AD) and/or major depressive disorder (MDD) from non-caseness in adolescent girls was better or equivalent to that of specialized instruments [26].

At first, the relationship between SOC scores and self-assessed symptoms of anxiety and depression were investigated by correlations and multiple regression models. Secondly, by using multivariate analyses, we investigated whether SOC and the measures of anxiety and depression separated themselves into distinct categories. Thirdly, we investigated whether the SOC score related to health parameters differently compared to measures of anxiety and depression. Finally, we compared the temporal stability of SOC (considered to measure trait) with the temporal stability of measures of anxiety and depression (considered to measure state).

## Method

### Samples

The non-clinical sample consisted of adolescent females (n = 66), with a mean age of 16.5 years (range 15.9-17.7 years). This sample was recruited from high schools in a small rural town, in Stockholm city, in an affluent northern suburb and in a less affluent southern suburb with a large immigrant population. Students received in oral and written information about the study. About 80 percent of the informed students participated, the participation ratio being similar for all schools. The main reasons for declining to participate were fear of blood sampling and reluctance to miss school-hours.

The sample of adolescent female psychiatric patients (n = 73) had a mean age 16.8 years (range 14.5-18.4 years) and had been diagnosed with of one or several of the following anxiety disorders (AD): general anxiety disorder (GAD), social anxiety disorder (SAD), specific phobia, panic disorder, separation anxiety, post-traumatic stress disorder (PTSD) and/or major depressive disorder (MDD). The subjects had ongoing treatment contact (median duration 11 months) at one of 13 open psychiatric clinics situated in the centre of Stockholm, its suburbs and in smaller towns nearby. One of the authors informed the staff at the clinics about the study and the staff then asked their patients about participation and gave them written information. According to staff reports 85 percent of the informed patients participated, the remaining number declined to do so out of fear of blood sampling or parents not approving the procedure. Assessment by child and adolescent psychiatrists or psychologist and a semi-structured diagnostic interview - Development and Wellbeing Assessment (DAWBA) - were used to establish the diagnosis of AD and/or MDD. Patients with severe autism or anorexia nervosa, mental retardation or psychotic symptoms were not considered for inclusion in the study. Two of the authors independently rated the computer-generated DAWBA information of all patients. In four cases the raters reported different diagnoses and in all of these cases the diagnostic dilemma was to differentiate GAD and MDD. However the raters reached consensus after careful assessment of the available information. Six subjects were denied participation because the DAWBA was incomplete or could not confirm diagnosis of AD and/or MDD. A detailed flow chart of the sampling procedure is previously published [27]. The study was approved by the Central Ethic's committee at Karolinska Institutet.

### Self-assessment questionnaires

**Sense of Coherence (SOC) **contains 29 items measuring putatively salutogenic factors [3,28]. Every item is rated on a 7-point scale giving a maximum score of 203. High scores indicate a good SOC. In a Swedish student population age < 30 years, the means were estimated to be 140 (SD 21.5) for women (N = 104) and 143 (SD 21.8) for men (N = 121) [29].

**Beck's Depression Inventory (BDI) **consists of 21 items rated on a 4-point scale and yields a total score by summation of the ratings for the individual items [30]. The total score ranges from 0-63 p and high scores indicate more severe depression. When this study was designed, the BDI-II had not yet been validated for the Swedish version and therefore BDI-A1 is used in this study.

**Beck's Anxiety Inventory (BAI) **contains 21 items assessing the degree to which the respondent has been affected by the physical or cognitive symptoms of anxiety during the past week [31]. BAI items are also meant to reflect panic attack symptoms. The total score ranges from 0-63 p and high scores indicate more severe anxiety.

**Strengths and Difficulties Questionnaire (SDQ) **is an internationally used screening instrument for mental health problems in children and teenagers [32]. It comprises 25 statements regarding psychological attributes and behaviours, forming five subscales. In this study, only the emotional subscale (SDQ-em) was used. Acceptable psychometric properties for the self-report version of SDQ for adolescents have been shown in previous Swedish studies [33].

**Psychosomatic health **was measured by frequency of having headaches, back pain, stomach problems and sleeping problems defined on a five-point scale by "never", "seldom", "1-2 days per week", "3-4 days per week", "every day".

**The level of subjectively perceived stress **in relation to total life situation, in relation to schoolwork and in relation to parents' life situation was assessed by a three-point scale defined by "never accurate", "sometimes accurate" and "always accurate".

**Sense of support **(by teachers and parents) **and sense of satisfaction **(likes to be in school and likes to be with friends) were assessed by a three-point scale defined by, "never accurate", "sometimes accurate", "always accurate".

**Health behaviors **were assessed by the frequency of physical activity (hard breathing, sweating), and going to bed after midnight ("never", "seldom", "once a week", "twice a week", ">twice a week") and by skipping breakfast and smoking of cigarettes ("never", "seldom", "1-2 d/week", "3-4 d/week" or "every day"). Estimated number of hours spent watching TV per week was also reported.

**Socio-demographic background **was assessed by two-alternative questions: "one or both parents born in Sweden/both parents born abroad", "living with both parents/living with *single parent", "both parents employed/one or both parents unemployed".*

The items of psychosomatic health, subjectively perceived stress, sense of support and satisfaction, health behaviors and socio demographic background did only address the present status.

### Diagnostic interview

**Development and Wellbeing Assessment (DAWBA**) is a semi-structured diagnostic interview designed to generate ICD-10 and DSM-IV psychiatric diagnoses on 5-17 year olds. DAWBA has consistently generated sensible estimates of prevalence and association with risk factors supporting good validity [34]. No published data are available on the inter-rater reliability of DAWBA, but when compared to non-manually based clinical diagnoses, DAWBA diagnoses support good validity [34-36]. In this study, the information was only collected from the patients and not from parents and teachers.

### Physiological health parameters

**Saliva cortisol **was collected on an ordinary school-day, the first sample shortly after waking up (still in bed), the second sample 30 min later. The Salivette sampling device with no preservative (Sarstedt) was used, the tube consisting of a plastic sampling vessel with a sterile neutral cotton wool swab, which had to be chewed for about 30 s and then returned to the insert. The subjects noted the time for each sample on the test-tubes and posted them to the laboratory. The saliva samples were stored at the laboratory at -20 C and analyzed by batch. The subjects were given both written and verbal instructions, and were requested not to collect saliva if they had a cold or were ill, and not to smoke cigarettes or use oral tobacco within two hours before sampling. Orion Diagnostica SPECTRIA^R ^Test Cortisol RIA, a test based on a competitive immunoassay principle, routinely used for quantitative in vitro estimation of cortisol in saliva, was used to determine the cortisol concentration in the saliva samples. The area under the curve between the first and second measurement in relation to baseline was calculated as a measure of the awakening response.

**Heart rate variability (HRV) **was measured with the subjects sitting upright, in silence, with no body movements allowed. None of the subjects had clinical signs or symptoms of infectious disease. Use of tobacco (oral tobacco and smoking of cigarettes) or intake of tea, coffee, caffeinated soft drinks or beta stimulant asthma medication was not allowed one hour prior to the measurements. The HRV registration was preceded by 15 min of rest. HRV was measured for 2 min × 2, in between which blood pressure was checked. This was a modified version of a 12 min protocol [37]. The standard deviation of inter-beat intervals (SDNN) was used as a time domain measure and high frequency and low frequency of HRV as frequency domain measures. In spectral analyses, variability distributes as a function of frequency [38]. High frequency HRV (0.15-0.4 Hz) is related to vagal activity and includes the respiratory sinus arrhythmia when the breathing rate is normal. Low frequency HRV (0.04-0.15 Hz) has been interpreted as reflecting both sympathetic and vagal input [39] but recent studies claim that low frequency mirrors mainly vagal influence [40].

**Plasma(p)-glucose **was analyzed with a portable Heamocue Glucose System device [41], the capillary sample being drawn right after the HRV measurement. The sample did not constitute a proper fasting sample.

**Weight and height **were measured and body mass index calculated (BMI = weight (kg)/height (m^2^)).

### Statistical Analyses

The relation between self-assessment scales and health variables were assessed by Pearson's product-moment correlations or with the Spearman rank test when these variables were of an ordinal nature. Partial correlations were used to remove the effect of heart rate, systolic-, diastolic blood pressure, body mass index, p-glucose and physical activity on HRV. Comparisons between two measurements were made in a two-tailed fashion with the paired sample t-test, or with Wilcoxon's sign ranks test when normal distributions were absent. Variables with a positively skewed distribution were logarithmically transformed. Logarithmically transformed HRV and cortisol parameters were normally distributed when the non-clinical and clinical samples were analyzed separately.

To assess the degree of prediction of BDI, BAI and SDQ-em respectively on SOC in the non- clinical and clinical samples a multiple regression model was used and of which the beta values were presented. By multiple regression analyses we could also evaluate the total effect of depressive, anxious and emotional symptoms on SOC. The multiple R^2 ^represents the coefficient of determination and has the disadvantage of increasing with the amount of predictors added. Therefore we also presented the adjusted R^2 ^[42]. Principal component analyses were used for orthogonal decomposition of the variables [43]. Explorative factor analyses and hierarchical cluster analyses were used to investigate whether the items of the scales arranged themselves in distinct categories [44]. Probability levels of 0.05 or less were considered significant and confidence intervals of 95% were reported. Analyses were done in Statistica 8.0 http://www.statsoft.com or SPSS 17.0 http://www.spss.com.

## Results

### Sample characteristics

The DAWBA interview concluded that 19.2 percent of the subjects fulfilled the criteria for only MDD, 32.9 percent for only one or several AD, and 47.9 percent received the combined diagnosis of both MDD and AD. The diagnosis of GAD constituted 34 percent and SAD 31 percent of the total amount of AD-diagnoses. Comorbidity of two or several AD occurred in 30.1 percent of the patients, while comorbidity with another psychiatric diagnosis in addition to AD and or MDD occurred in 37.0 percent of the patients. The group with other psychiatric diagnoses in addition to AD and/or MDD did not show extreme scores on any of the assessment scales. On the contrary, they scored lower than the group with comorbidity of AD and MDD (data not shown).

### SOC versus self-assessment of anxiety and depression

The internal consistency for SOC, BDI, BAI and SDQ-em were high in both samples as described by Cronbach's alpha (table 1). SOC showed the highest negative correlations to the BDI in the non-clinical sample on both measurements and also in the clinical sample (table 2). Multiple regression models showed that BDI was the strongest predictor of SOC in the non-clinical (beta coefficient -0.47) and clinical sample (beta coefficient -0.52) (table 3). Multiple regression analyses also showed the degree of explanation of self assessed anxiety and depression (BDI, BAI and SDQ-em) on the SOC variance in the non-clinical sample, estimated by multiple R^2 ^= 0.74, adjusted R^2 ^= 0.73 and in the clinical sample multiple R^2^= 0.66, adjusted R^2 ^= 0.65.

**Table 1:** Cronbach's alpha for the Sense of Coherence, Beck's Depression Inventory, Beck's Anxiety Inventory and the emotional subscale of Strength's and Difficulties questionnaire

	SOC	BDI	BAI	SDQ-em
Non-clinical sample	0.94	0.87	0.93	0.71
Clinical sample	0.94	0.91	0.93	0.56

**Table 2:** Spearman's rho correlations of SOC, BDI, BAI and SDQ-em scores in measurement 1 of the non-clinical sample (NC1), measurement 2 of the non clinical sample (NC2) (6 months interval) - and in the clinical sample (C).

	*NC-BDI 1*	*NC-BAI 1*	*NC-SDQ-em 1*	*NC-BDI 2*	*NC-BAI 2*	*NC-SDQ-em 2*	*C-BDI*	*C-BAI*	*C-SDQ-em*
* NC-SOC 1*	-0.86*** N = 50	-0.78*** N = 50	-0.73*** N = 50						
*NC-BDI 1*		-0.80*** N = 66	-0.73*** N = 66						
*NC-BAI 1*			-0.73*** N = 66						
*NC-SOC 2*				-0.79*** N = 59	-0.65*** N = 59	-0.71*** N = 59			
*NC-BDI 2*					-0.75*** N = 62	-0.78*** N = 62			
*NC-BAI 2*						-0.67*** N = 62			
*C-SOC*							-0.74*** N = 64	-0.70*** N = 68	-0.53*** N = 69
*C-BDI*								-0.67*** N = 66	-0.44*** N = 67
*C-BAI*									-0.50*** N = 70

*** significant at the p < 0.001 level

**Table 3:** General regression model showing the degree of prediction of BDI, BAI and SDQ-em on SOC in the non-clinical and clinical sample.

*Assessment scale*	*Non-clinical sample*	*Clinical sample*
	***Beta (CI)***	***Beta (CI)***
	
* BDI*	*-0.47 (0.75 to -0.19) N = 50*	*-0.52 (-0.71 to -0.32) N = 64*
*BAI*	*-0.18 (-0.45 to 0.08) N = 50*	*-0.23 (-0.43 to 0.02) N = 68*
*SDQ-em*	*-0.28 (-0.51 to -0.04) N = 50*	*-0.22 (-0.39 to -0.04) N = 69*

### Multivariate analyses on item level

**Figure 1:**
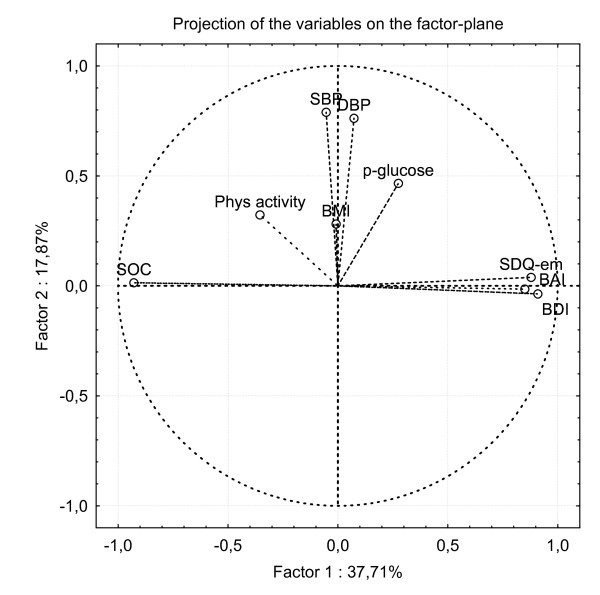
The projection of the scores of SOC, the psychiatric assessment scales (BDI, BAI, SDQ-em) and physiological health-related variables (systolic blood pressure SBP, diastolic blood pressure DBP, physical activity and  plasma-glucose) on the factor plane calculated by principal component analysis.

**Figure 2:**
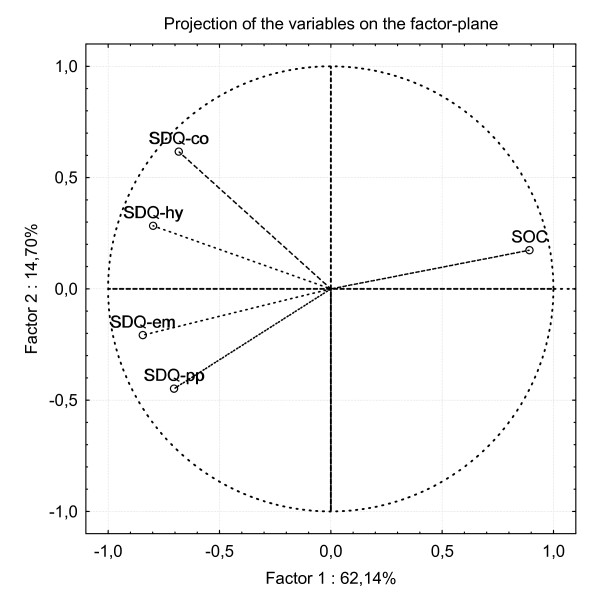
The projection of the scores of SOC and the subscales of SDQ (emotional, peer problems, conduct problems, hyperactivity) on the factor plane calculated by principal component analysis.

All multivariate analyses were performed on the combined samples. Explorative factor analyses failed to identify SOC as a distinct construct separated from the anxiety and depression constructs. Principal component analyses (PCA) showed an orthogonal decomposition in which SOC versus BDI, BAI and SDQ-em projected themselves in the factor plane opposite each other (figure 1). Furthermore, principal component analysis including the SOC and the SDQ subscales of emotional problems, peer problems, conduct problems and hyperactivity clearly demonstrated that only SDQ-em and SOC have the same dimensionality as opposed to peer problems, conduct problems and hyperactivity that have unique dimensionality compared to SOC (figure 2).

Hierarchical cluster analyses solely applied to SOC items did not confirm any categories of meaningfulness, manageability and comprehensibility. Hierarchical cluster analysis performed on all items from all the scales revealed that 17 of the BAI-items and one SDQ-em item that addressed severe anxiety and physiological reactions of fear, constituted a separate cluster. All SOC and BDI items remained in the other cluster (data not shown).

### SOC, BDI, BAI and SDQ-em versus health parameters

Generally SOC, BDI, BAI and SDQ-em showed a similar pattern of correlation to both self-reported and physiological health related parameters, although SOC often showed higher correlations. Among the physiological parameters, only the awakening response of saliva cortisol and the high frequency HRV correlated to SOC, BDI, BAI and SDQ-em in the non-clinical sample and the correlations were strongest for SOC. The correlations between SOC and the self-assessed health-related parameters were generally lower in the clinical sample than the non-clinical sample (table 4).

**Table 4:** Showing Pearson correlation coefficients calculated with pair-wise exclusion between SOC, BDI, BAI and SDQ-em and self-assessed and physiological health-related parameters in the non-clinical (N = 66) and clinical sample (N = 73).

Non-clinical sample						Clinical sample						
Parameter	N	Mean (SD)	SOC N = 55	BDI N = 66	BAI N = 66	SDQ-em N = 66	N	Mean (SD)	SOC N = 73	BDI N = 67	BAI N = 70	SDQ-em N = 73
SOC	55	137 (27)					73	96 (20.5)	97			
**Psychiatric symptoms**^**1**^												
Depressive symptoms BDI	66	9.8 (8.4))	-0.86***	-	0.80***	0.73 ***	67	25.1(12.0)	-0.74***	-	0.67***	0.44***
Anxiety symptoms BAI	66	13.3 (9.7)	-0.78***	0.79***	-	0.73***	70	22.8(11.0)	-0.70***	0.67***	-	0.50***
Emotional problems SDQ-em	66	3.7 (2.4)	-0.73***	0.73***	0.73***	-	73	3.7 (2.3)	-0.53***	0.44***	0.50***	-
Hyperactivity SDQ-hy	66	3.7(2.6)	-0.62**	0.64**	0.66**	0.53**	73	3.7(2.5)	-0.52***	0.51***	0.51***	0.37**
Conduct problems SDQ-co	66	1.3(1.3)	-0.52**	0.58**	0.59**	0.53**	73	1.3(1.3)	-0.40*	0.42***	0.46***	0.21
Peer problems SDQ-pp	66	1.5(1.8)	-0.62**	0.53**	0.57**	0.38*	73	1.5 (1.8)	-0.33**	0.36**	0.17	0.21
**Psychosomatic symptoms**^**1**^												
Headache	66	2.5(0.9)	-0.42**	0.40**	0.50**	0.59**	70	3.1 (1.0)	-0.26*	0.26*	0.40**	0.37**
Backache	66	2.6 (1.0)	-0.54**	0.51**	0.54**	0.48**	70	3.1 (1.2)	-0.25*	0.19	0.27*	0.39**
Stomach problems	65	2.3 (0.8)	-0.39**	0.25*	0.33*	0.28*	70	3.5 (1.2)	-0.19	0.18	0.25*	0.23
Sleep problems	66	2.6 (1.0)	-0.45*	0.54**	0.61**	0.53**	70	2.6 (1.2)	-0.25*	0.51**	0.30*	0.11
Dizziness	66	1.9(0.9)	-0.26	0.32**	0.41**	0.09	70	2.0 (0.8)	-0.43**	0.34**	0.46**	0.31**
**Self-perceived stress**												
In relation to total life situation	64	2.6 (0.6)	0.41**	-0.56**	-0.50**	-0.33*	67	2.0 (0.8)	0.19	-0.23	-0.06	-0.13
In relation to school work	66	1.6 (0.6)	0.51**'	-0.42**	-0.32**	-0.40**	70	1.4 (0.6)	0.17	-0.14	-0.08	-0.11
In relation to parent's situation	66	2.2 (0.6)	0.26	-0.34**	-0.24*	-0.18*	66	1.9 (0.8)	0.12	-0.18	-0.28*	-0.06
**Sense of support/satisfaction**^**1**^												
By teachers	66	1.6 (0.6)	0.55**	-0.47**	0.46**	-0.42**	70	2.0 (0.6)	0.55**	-0.37**	-0.39**	-0.22
By parents	66	1.2 (0.7)	0.43**	-0.38**	0.40**	-0.38**	70	1.4 (0.7)	0.21	-0.20	-0.22	-0.35**
Likes to be in school	66	1.7 (0.7)	0.48**	-0.47**	0.40**	-0.33*	66	2.3 (0.6)	0.11	-0.08	-0.12	0.03
Likes to be with friends	66	1.3 (0.5)	0.23	0.24	0.14	0.20	66	1.9 (0.7)	0.00	-0.01	0.10	0.13
**Health behaviours**^**1**^												
Physical activity	66	3.5(1.1)	0.11	-0.04	0.09	-0.07	70	3.0(1.3)	0.24*	-0.31*	-0.29*	-0.18
Skips breakfast	66	2.4(1,2)	-0.32*	0.22	0.29*	0.30*	70	3.0(1.3)	-0.24	0.30*	0.20	0.04
TV hours	62	4.7 (2.7)	-0.12	0.11	0.15	0.26*	69	4.3(2.9)	0.22	-0.17	-0.13	-0.16
Daily smoking	61	1.8 (1.3)	-0.34*	0.17	0.20	0.24*	69	2.1(1.3)	-0.18	0.04	0.20	0.08
**Objective health parameters**^**2**^												
Body Mass Index	66	22.2(3.6)	-0.08	0.04	0.04	0.02	67	21.3 (3,9)	-0.04	-0.01	-0.10	0.06
P-glucose	66	5.5 (0.7)	-0.07	0.17	-0.04	0.01	69	6.7 (2.2)	0.07	-0.04	0.10	-0.01
Saliva cortisol AUC-b	46	2.1 (0.3)	-0.32*	0.27	0.21	0.14	35	1.7 (0.7)	-0.30	0.18	0.04	0.22
Blood pressure systolic	65	111 (9.7)	0.03	0.17	0.14	0.00	69	109 (16.6)	-0.15	0.01	-0.01	0.20
Blood pressure diastolic	65	67 (7.5)	0.07	-0.06	-0.08	-0.15	68	68 (9.2)	0.06	-0.01	-0.02	0.09
HRV high frequency (HF)	53	5.9 (0.83)	0.32*	-0.19	-0.16	-0.15	60	5.5 (0.86)	-0.09	0.03	-0.05	0.09
HRV low frequency (LF)	53	5.9 (0.87)	0.15	-0.06	-0.06	-0.06	60	5.5 (0.86)	-0.04	0.01	-0.04	0.06
HRV st d of inter-beat int (SDNN)	53	4.1 (0.32)	0.20	-0.14	-0.07	-0.08	60	3.88 (0.34)	-0.13	0.04	0.01	0.11
HRV HF adjusted for HR	42	5.9 (0.84)	0.41**	-0.32*	-0.30	-0.43**	49	5.4 (0.88)	-0.05	0.06	0.03	0.17
HRV LF adjusted for HR	42	6.0 (0.90)	0.17	-0.20	-0.13	-0.28	49	5.5 (0.91)	0.01	0.04	0.04	0.08
HRV SDNN adjusted for HR	42	4.1 (0.33)	0.28	-0.35*	-0.28	0.44**	49	3.88 (0.35)	-0.09	0.08	0.12	0.17
**Socio-demographic factors**^**1**^												
Parent unemployment	65	25%	-0.15	0.15	0.22	0.25*	70	31%	0.03	0.02	0.07	0.14
Parent non Swedish ethnicity	66	24%	-0.02	0.08	0.14	0.14	70	6%	0.15	0.00	-0.05	-0.29*
Single parent family	62	27%	0.06	-0.02	-0.07	0.06	70	47%	0.09	-0.09	-0.02	-0.28*

*** significant at the p < 0.001 level, ** p < 0.01, * p < 0.05

^1 ^Spearman rank correlations, ^2 ^Pearson product correlations, ^3^When adjustments were done also for systolic blood pressure SBP, diastolic bloodpressure DBP, body mass index, p-glucose and physical activity the significant correlations between HF and SDNN and self-assessment scales remained (SOC: HF 0.42**,SDNN 0.24 ns; BDI: HF -0.36**, SDNN-0.32**, BAI: HF -0.36*, SDNN -0.29 ns, SDQ-em: HF -0.40*, SDNN -0.33*)

### Temporal stability

The highest correlations between the first and second measurement were found for SOC followed by BDI, BAI, SDQ-em (table 5). The Wilcoxon matched pair test showed significant differences of BDI and BAI, but not of SOC and SDQ-em, between the measurements (table 5). The correlations between the first and second measurement were higher for all assessment scales in the low SOC-score quartile of the non-clinical sample compared to the high SOC-score quartile (data not shown). BDI and BAI showed minor variation of over time, but showed significant correlation to SOC on both measurements (table 2).

**Table 5:** Correlations and paired sample t-tests of self assessment scores comparing measurement 1 and 2 (6 months interval). (Wilcoxon sign rank test is provided in addition since normal distribution was not present in all cases).

	N	Mean (SD)	**Corr. Coeff**^**1**^	**t-value**^**2**^	**Z-value**^**3**^
SOC 1	46	138.3 (27.8)			
SOC 2	46	138.5 (27.6)	0.90***	-0.15 ns	-0.03 ns
BDI 1	62	9.9 (8.7)			
BDI 2	62	7.8 (7.7)	0.84***	3.5**	3.29**
BAI 1	62	13.8 (9.8)			
BAI 2	62	10.2 (8.7)	0.83***	5.1***	4.87***
SDQ-em 1	62	3.63 (2.4)			
SDQ-em 2	62	3.39 (2.3)	0.72***	1.1 ns	0.89 ns

*** significant at the p < 0.001 level, **p < 0.01

^1^Pearson product correlations, ^2^Paired sample t-test, ^3^Wilcoxon signed ranks test

## Discussion

The main finding of this study was that the SOC scale appears to be an inverse measure of persistent and generalized symptoms of anxiety and depression. The SOC scale and self-assessed symptoms of anxiety and depression showed high correlations and multiple regression models showed that symptoms of anxiety and depression explained a major part of the SOC variance in both the non-clinical and clinical samples. The SOC scale and measures of anxiety and depression showed similar patterns of correlations to health-related parameters in both non-clinical and clinical samples of adolescent girls, similar to what has been shown in adults [10]. Multivariate analyses failed to isolate SOC as a separate construct distinct from measures of anxiety and depression. As the SOC items pertaining to the putative categories of meaningfulness, manageability and comprehensibility showed high covariance, the multivariate analyses failed to identify these as separate clusters. Previous factor analyses of SOC items in samples of Swedish students show similar results [29].

Regarding temporal stability, the highest correlations between the first and the repeated measurements six months later, were found for SOC followed by BDI, BAI, SDQ-em. This may be explained by the fact that the BDI and BAI ask about symptoms during the last two weeks. BDI and BAI may thus capture mood swings and shorter episodes of major depressive disorder and situational anxiety on top of more persistent depressive symptoms and generalized anxiety. Contradictory to the salutogenic theory [1] the low quartile of the SOC score in the non-clinical sample showed higher temporal stability than the high quartile (data not shown). The data failed to support that the SOC-scale is more stable at the high end of the continuum. A limitation of this investigation was the lack of repeated measures of the clinical sample, which would have given information of temporal stability in the very low end of the SOC continuum.

The extended hierarchical cluster analyses, that included all the items of SOC, BDI, BAI, SDQ-em, revealed that BAI and SDQ-em items that assessed symptoms of severe anxiety and physiological reactions of fear clearly separated themselves from the BDI and SOC items. It thus appeared as if BAI did not capture the type of anxiety typical for GAD or generalized SAD. The generalized type of anxiety was better identified by the SOC-scale. The results of the hierarchical cluster analyses cannot be regarded as evidence, but aid an alternative interpretation of SOC. The superior sensitivity of the SOC scale for caseness of emotional disorders in adolescent females described in our previous work [45] may be explained by the fact that the SOC scale covers symptoms congruent with the DSM-IV criteria for MDD, dysthymic disorder, GAD and generalized SAD.

The question of item-overlap between SOC and measures of anxiety and depression has previously been suggested [7] since meaninglessness/hopelessness is one of the cardinal symptoms of major depressive disorder. Furthermore, when suffering from MDD or generalized anxiety the cognitive function and social drive decrease leading to a diminished comprehensibility and manageability. In other psychiatric disorders such as ADHD, conduct disorder or situational anxiety this is not necessarily the case. However, comorbidity is common in this age group and depressive and anxious problems in combination with ADHD, conduct disorder or situational anxiety may explain a possible decrease of SOC and also the poorer outcome related to low SOC reported for ADHD [46].

In adolescence, a decline in social engagement can be the result of different trajectories. For example, depressive and anxious symptoms may co-exist and develop simultaneously to disorders of depression and anxiety. Alternatively, a primary diagnosis of SAD or GAD may lead to secondary depressive symptom. Finally, as often the case, primary MDD or dysthymic disorder generate secondary social problems. The differential diagnosing of MDD, GAD and SAD is specifically difficult in adolescence, since the diagnoses are highly co-morbid [47]. Genetic studies even indicate that depression and anxiety disorders may share a genetically determined neurobiological component [48,49]. Comorbidity tends to generate higher severity scores in adolescent girls [45] and comorbidity of GAD and MDD, is related to an increase of overall mortality in adults [50]. Adolescents with comorbidity of generalized anxiety and depression thus need to be identified and prioritized for treatment and deserve also more attention in future research.

The SOC-scores showed higher correlations to the awakening response of saliva cortisol compared to the psychiatric self-assessment scales in both samples. Due to the great loss of cortisol samples especially in the clinical sample this data is unsecure, nevertheless the finding is in line with our hypothesis that SOC but not BDI and BAI measures generalized anxiety, since in adolescents, persistent anxiety, but not current or situational anxiety, is associated with increase of the awakening response of saliva cortisol [51].

Earlier population-based and clinical studies have shown that a decrease of HRV is present both in anxiety and depression [52-54], although the correlation of HRV and SOC-score is not previously shown. In line with previous discussion the correlation between HRV and SOC support that autonomous regulation is impaired in adolescent girls with MDD, dysthymic disorder, GAD or generalized SAD.

The loss of SOC data (11 cases in the non-clinical sample) was due to incomplete forms from one of the schools at the first measurement and can be considered a random error. When omitting the subjects with incomplete forms the rest of the sample showed a strong correlation to the measures of anxiety and depression. The correlation was similar in repeated measures six months later when the full sample was included. The mean SOC score from the subjects from measurement 1 (mean 137.1 SD 26.9) and from the measurement 2 (mean 138.1 SD 27.5) were similar. Hence, the impact of this data loss did not seem to affect the conclusion. The loss of HRV data was due to registration artifacts caused by body movements was also random and should not have affected the conclusions.

The loss of salivary cortisol on the contrary must be regarded as a non-random error since it was more frequent in the clinical sample (non clinical 20/66 and in the clinical 38/73) creating an asymmetric loss in the samples. The loss of cortisol data may be linked to the depressed mood of the patients. However, the primary aim of including the salivary cortisol in table 1 was to compare the correlation between AUC-b cortisol and SOC, BDI, BAI and SDQ-em respectively.

## Conclusions

The SOC-scale appears to be an inverse measure of persistent depressive symptoms and generalized anxiety when applied to adolescent girls rather than a measure of a specific salutogenic construct. The symptoms captured by the SOC scale are similar to the diagnostic criteria for MDD, dysthymic disorder, GAD and SAD according to DSM-IV. These disorders are not adequately identified by the specialized self-assessment scales for anxiety and depression that are currently available in validated Swedish versions.

We can no longer rely on the assumptions that low SOC is a trait measure from late adolescence and that it measures a salutogenic construct separated from anxiety and depression. On the contrary active identification of adolescent girls with MDD, dysthymic disorder, GAD and SAD should be emphasized. Comorbidity of these disorders corresponds to increased symptom severity and high negative impact on quality of life and global functioning. Future research should aim to identify individuals with increased vulnerability for depressive and anxious problems and try out preventive methods. Early identification and treatment of depressive and anxious problems may prevent recurrent episodes and life-long suffering.

## List of abbreviations

BAI: Beck's Anxiety Inventory; BDI: Beck's Depression Inventory; DAWBA: Development and Wellbeing Assessment; GAD: Generalized Anxiety Disorder; HPA: Hypothalamic-Pituitary-Adrenal; HRV: Heart Rate Variability; MDD: Major Depressive Disorder; SAD: Social Anxiety Disorder; SDNN Standard Deviation of Inter Beat Intervals; SDQ-em: Strengths and Difficulties Questionnaire-emotional subscale; SOC: Sense of Coherence

## Competing interests

The authors declare that they have no competing interests.

## Authors' contributions

All authors contributed to and have approved the final manuscript. EHB was the "project leader" of the study, responsible for the data collection and wrote the first draft of the manuscript. ES contributed with recruitment and diagnostic issues of the clinical sample. TT contributed to the original outlines of the project and was responsible for the saliva cortisol analyses. JOL was responsible for psychometric references and literature search. MI was responsible for the over-all design of the study and for methodological issues.
